# Exosomes of A549 Cells Induced Migration, Invasion, and EMT of BEAS-2B Cells Related to let-7c-5p and miR-181b-5p

**DOI:** 10.3389/fendo.2022.926769

**Published:** 2022-07-08

**Authors:** Yun Liu, Chao-Yue Su, Yan-Yan Yan, Jian Wang, Jia-Jun Li, Ji-Jun Fu, Yu-Qing Wang, Jian-Ye Zhang

**Affiliations:** ^1^ Guangzhou Municipal and Guangdong Provincial Key Laboratory of Molecular Target and Clinical Pharmacology, the NMPA and State Key Laboratory of Respiratory Disease, School of Pharmaceutical Sciences and the Fifth Affiliated Hospital, Guangzhou Medical University, Guangzhou, China; ^2^ School of Medicine, Shanxi Datong University, Datong, China

**Keywords:** exosomes, lung cancer, migration, invasion, EMT, Let-7c-5p, MiR-181b-5p

## Abstract

As carriers containing abundant biological information, exosomes could deliver the property of donor cells to recipient cells. Emerging studies have shown that tumor cells could secrete a mass of exosomes into the microenvironment to regulate bystander cells. However, the underlying mechanisms of such a phenomenon remain largely unexplored. In this research, we purified and identified the exosomes of A549 cells and found that A549-cell-derived exosomes promoted BEAS-2B cells migration, invasion, and epithelial–mesenchymal transition (EMT). Importantly, we observed that let-7c-5p and miR-181b-5p were attenuated in A549-cell-derived exosomes compared to BEAS-2B-cell-derived exosomes. The analysis of miRNA expression level in BEAS-2B cells indicated that incubation with A549-cell-derived exosomes reduced the expression levels of let-7c-5p and miR-181b-5p. In transient transfections assay, we found that downregulation of let-7c-5p and miR-181b-5p simultaneously showed stronger promotion of BEAS-2B cells migration and invasion than individually. Moreover, exosomes secreted from A549 cells with upregulated expression of let-7c-5p and miR-181b-5p significantly reduce their regulatory effect on BEAS-2B cells. Bioinformatics analyses revealed that let-7c-5p and miR-181b-5p inhibit the EMT process mainly by regulating focal adhesion and mitogen-activated protein kinase (MAPK) signaling pathway. Thus, our data demonstrated that A549-cell-derived exosomal let-7c-5p and miR-181b-5p could induce migration, invasion, and EMT in BEAS-2B cells, which might be regulated through focal adhesion and MAPK signaling pathway. The expression level of let-7c-5p and miR-181b-5p may show great significance for the early diagnosis of lung cancer.

## Introduction

As the leading cause of death, cancer threatens the health of humanity worldwide. According to the Global Cancer Statistics 2020 reports, lung cancer was one of the most commonly diagnosed cancers among various cancer types, with an estimated 2.24 million new cases (11.4%) and 1.8 million deaths (18%), showing that lung cancer was the leading cause of cancer-related deaths in 2020 (1). Although the diagnosis and treatment of lung cancer get more precise for the past few years, tumor metastasis remains the biggest obstacle to treatment efficacy and prognosis of lung cancer, resulting in high rates of deaths (2). Thus, further investigation of lung cancer pathogenesis and metastasis mechanism is crucial.

Epithelial–mesenchymal transition (EMT) is a widespread physiological and pathological reversible dynamic process of growth, development, and disease (3). During EMT, cytoskeleton and characteristics of epithelial cells are changed along with the drop of epithelial surface markers, for instance, E-cadherin. After EMT, epithelial cells acquire a mesenchymal phenotype and lessen intracellular adhesion and proliferative capacity, which in turn enhances the invasion and migration of cancer cells (4). A plethora of studies indicate that exosomes take part in the EMT process of lung cancer by transferring mesenchymal-induced signals, which induce cancer cells to more likely migrate to distant sites (5–7). Exosomes are biological vesicles that contain a large number of biologically active molecules, such as proteins, mRNAs, and miRNAs (8). As exosomes carry various biological molecules into the recipient cells, the latter can obtain the specificity of the donor cells (9). Furthermore, tumor-cell-derived exosomes are associated with the origin, proliferation, and invasion of tumors, which may induce normal cells to acquire relevant tumor characteristics and malignant transformation (10). The aberrantly expressed tumor-cell-derived exosomal miRNA can affect cancer progression and metastasis (11; 12). EMT is an important process of the early stage of tumor initiation, which is closely associated with tumor growth, metastasis, and angiogenesis. Tumor cells undergoing EMT will enhance the ability of migration and invasion (13).

Our previous work showed that attenuation of exosomal miR-34c-3p promotes invasion and metastasis of NSCLC by altering integrin α2β1. Herein, we had isolated and identified tumor-cell-derived exosome to investigate its influence on EMT, migration, and invasion of normal epithelial cells and the vital biomolecule function during this process.

## Materials and Methods

### Cell Culture and Reagents

Two cell lines were used in this study, namely, A549 cells (an adenocarcinoma human lung epithelial cell line) and BEAS-2B cells (a human bronchial epithelial cell line), which were obtained from iCell Bioscience Inc. (Shanghai, China). Both cell lines have acquired short tandem repeats (STRs). A549 cells were cultured in Ham’s F-12K (Kaighn’s) medium, and BEAS-2B cells were in Dulbecco modified Eagle’s medium (DMEM), both supplemented with 10% exosome-depleted fetal bovine serum EXO-FBS-50A-1 (System Biosciences, Palo Alto, CA) to avoid interference from bovine exosomes and with 1% penicillin–streptomycin solution (Tianhang Biotechnology, Hangzhou, China). Cells were incubated at 37°C and in a 5% CO_2_ atmosphere (14).

### Exosomes Isolation

A549 cells were planted and well attached overnight; the cell culture medium was acquired 48 h later. Exosomes were isolated using differential centrifugation as described (15; 16). The medium was centrifuged at 300*g* for 10 min to deplete residual cells. Supernatants were collected and centrifuged at 2,000*g* for 10 min to eliminate cells debris. The supernatant was filtered through a 0.22-μm filter (EMD Millipore Corporation, Darmstadt, Germany) to remove apoptotic bodies and microvesicles; then, the exosomes were precipitated by ultracentrifugation at 100,000*g* for 90 min at 4°C. Finally, pellets were resuspended washed in phosphate-buffered saline (PBS) and centrifuged again at 100,000*g* for another 90 min.

### Exosomes Identification

The prepared exosomes were visualized by transmission electron microscopy (TEM) (17). Briefly, exosomes were resuspended in PBS after being centrifuged, then were dropped in a carbon-coated copper grid and incubated with 2% uranyl acetate. Images were acquired using JEM-1400 operated at 80.0 kV. The size distribution analysis of exosomes was performed on NanoSight Nanoparticle Tracking Analysis (NTA) systems (NTA 3.0., Malvern Instruments, UK) configured with a high-sensitive sCMOS camera. Prepared exosomes were manually loaded into the sample chamber at environment temperature (18). Each sample needed to be measured in triplicate. The exosomes surface markers were identified by Western blotting analysis for β-catenin (Cell Signaling Technology, Inc., MA, USA), CD81 (Abcam Biotechnology, CA), TSG101(Invitrogen, Carlsbad, CA, USA), and CD63 (AbcamBio Technology, CA).

### Clinical Serum Samples

Healthy persons (n =21) and non-small cell lung cancer (NSCLC) patients (n =37) from the Second People’s Hospital (Datong, China) were recruited in this study. All serum samples were collected for insolation of exosomes. The study protocol was approved by the ethical review committees of the Second People’s Hospital of Datong. Informed consent forms were given to patients signing for confirmation of the sample collection.

### Wound Healing Assay

The *in vitro* wound healing assay was used to measure the mobility of BEAS-2B cells, after treatment with the isolated A549-cell-derived exosomes as described previously (14). Briefly, cells were collected, and the resuspension was filtered by a cell strainer to produce single-cell suspension. After adhering for overnight in six-well plates, the cells that had reached confluence were affirmed. Straight scratch was processed by a 10-μl pipette tip on the monolayer cells. PBS was utilized to wash out the loosing cells gently. The cells were incubated with different concentrations of exosomes (15, 30, and 60 μg/ml), respectively. Wound healing images were captured at different treatment times of exosomes. The repaired area was measured to compute the cell motility according to the healing percentage.

### Invasion Assay

Transwell chambers (Greiner Bio-One, Frickenhausen, Germany) were applied to produce the invasion ability of cells. The procedure was performed routinely as previously described (16). The Matrigel (Corning Costar, Corning, NY) mixture with medium was first coated into the apical chamber; then, 2×10^5^ cells were seeded. The complete medium containing the specified concentration of exosomes (0, 30, and 60 μg/ml) were added to basolateral chambers as chemoattractant. After cells had invaded for 24 h, methanol was adopted to fix the upper chamber before staining with 0.05% crystal violet. Image of the migrated cells was captured by a microscope (Olympus Life Science, Tokyo, Japan). More than nine images of random fields were selected for quantification. IMAGE-J software (http://imagej.nih. gov/ij/) was used for quantification, and then, the comparison of migrated cells and total attached cells for each group were analyzed.

### Western Blotting

As our previous report described (19), briefly, pretreated cells were collected and washed twice with ice-cold PBS and then mechanically lysed in radioimmunoprecipitation assay (RIPA) buffer (Thermo Fisher Scientific, Waltham, MA, USA), supplemented with premixed reagent Proteinase Inhibitor (Cell Signaling Technology, Danvers, MA, USA) and phenylmethylsulfonyl fluoride (PMSF, Cell Signalling Technology, Danvers, MA, USA). The concentration of purified proteins was quantified by bicinchoninic acid (BCA, Thermo Fisher Scientific, Waltham, MA, USA) protein assay kit.

Proteins of equal quality (50 μg/lane) were separated on a sodium dodecyl sulfate–polyacrylamide gel electrophoresis (SDS-PAGE) by electrophoresis and blocked after transferring to polyvinylidene fluoride (PVDF) membranes (Millipore, Boston, MA). Then, blots were incubated with primary antibodies overnight according to the protocol. Corresponding horseradish peroxidase (HRP)-conjugated secondary antibodies (1:10,000, Cell Signaling Technology, USA) were treated for 2 h. Blots were revealed by Western Lightning^®^ Plus-ELC kit (PerkinElmer, Ma, USA) and exposed by ChemiDoc™ XRS+ (Bio-Rad, CA, USA). The included antibodies were as follows: the primary antibodies β-catenin (1:1,000, Cell Signaling Technology, USA), HSP70 (1:2,000, Bioworld Technology, China), TSG101 (1:1,000, Cell Signaling Technology, USA), CD63 (1:1,000, Abcam, USA), and CD81 (1:1,000, Abcam, USA), and the secondary antibodies HRP anti-mouse (1:10,000, Bioworld Technology, China) and anti-rabbit (1:2,000, Cell Signaling Technology, USA).

### Transient Transfections

miRNA mimics and inhibitors [negative control (NC), let-7c-5p mimic/inhibitor and miR-181-5p mimic/inhibitor] used in transient transfections were synthesized by GenePharma, Inc. (Shanghai, China). Experimental procedure followed the manufacturer’s instructions. For each transfected sample, the RNA was diluted with 50 μl Opti-MEM and mixed by gently pipetting three to five times, and 1.0 μl Lipofectamine 2000 (Invitrogen) was diluted with 50 μl Opti-MEM. Then, the above two reagents were mixed in a concentration of 50 nM and incubated at room temperature for 20 min. After BEAS-2B cells had reached 70% confluence, 100 μl per well of transfection complex was added. Cells were harvested for subsequent studies at 24/48 h post-transfection.

### RNA Preparation and RT-qPCR Assays

An Exosomes RNA Isolation Kit (System Biosciences, Palo Alto, CA) was used to isolate and concentrate the exosomes containing RNA according to the manufacturer’s instructions. The total RNA of A549-cell-derived exosomes (15, 30, and 60 µg/ml) post-treated BEAS-2B cells at 24 h was isolated by using Trizol (Thermo, MA, USA) followed by purification with isopropanol and ethyl alcohol. RNase-free water was used to dissolve the isolated RNA; then, quantified measurement was perform by Thermo Scientific™ Nanodrop2000 platform (Thermo Fisher Scientific, Waltham, MA, USA). The Mir-X™ miRNA qRT-PCR SYBR Kit (TaKaRa, Japan) was adopted to synthesize complementary DNA (cDNA) with equal amount of miRNA (or RNA). Relevant RNA levels were assessed by Agilent StrataGene Mx3000P qPCR (Agilent Technologies, USA) with SYBR^®^ Green Real-Time PCR Master Mix (Toyobo, Osaka, Japan) and were analyzed using the 2^−ΔΔCt^ method (20). The sequences of mRNA primers that have been implicated in our research are exhibited in **Table 1**. Quantification of miRNA were conducted using a forward primer (TaKaRa, Japan) that was designed to detect mature hsa-Let-7c-5p and miR-181b-5p. The miRNA primer sequences that were employed in the quantification are listed in the **Table 1**. The universal primer included in the kit was applied as the reverse primer, and U6 was recruited as the internal reference for adjusting miRNA expression.

**Table 1:** The sequences of mRNA/miRNA primers.

mRNA/miRNA	forward primer	reverse primer
N-Cadherin	CGAATGGATGAAAGACCCATCC	GCCACTGCCTTCATAGTCAAACACT
E-Cadherin	AGGATGACACCCGGGACAAC	TGCAGCTGGCTCAAGTCAAAG
Vimentin	AGGATGACACCCGGGACAAC	TGCAGCTGGCTCAAGTCAAAG
GAPDH	GGAAGGTGAAGGTCGGAGTCA	GTCATTGATGGCAACAATATCCACT
hsa-let-7c-5p	TGAGGTAGTAGGTTGTATGGTT	CAGTGCGTGTCGTGGAGT
hsa-miR-17-3p	GCAGTGAAGGCACTTGTAG	CAGTGCGTGTCGTGGAGT
hsa-miR-92b-3p	CACTCGTCCCGGCCTCC	CAGTGCGTGTCGTGGAGT
hsa-miR-92b-5p	ACGGGACGCGGTGCAGTG	CAGTGCGTGTCGTGGAGT
hsa-miR-155-5p	TAATGCTAATCGTGATAGGGG	CAGTGCGTGTCGTGGAGT
hsa-miR-181b-5p	ATTCATTGCTGTCGGTGGGT	CAGTGCGTGTCGTGGAGT
hsa-miR-200b-3p	TAATACTGCCTGGTAATGATGA	CAGTGCGTGTCGTGGAGT
hsa-miR-224-5p	GTCACTAGTGGTTCCGTTTAG	CAGTGCGTGTCGTGGAGT
U6	CGCAAGGATGACACG	GAGCAGGCTGGAGAA

### Target Genes Prediction

To investigate the potential target pathway of let-7c-5p and miR-181b-5p, bioinformatics analyses were subsequently utilized for the research. Briefly, TargetsScan (http://www.targetscan.org/mamm_31/), PicTar (https://pictar.mdc-berlin.de/cgi-bin/PicTar_vertebrate.cgi), and miRDB (http://mirdb.org/mirdb/index.html) were applied to obtain the target of the subject miRNA. The relative genes of NSCLC were obtained from the following databases: GeneCards (https://www.genecards.org/), Disgenet (https://www.disgenet.org/), TTD (http://db.idrblab.net/ttd/), and OMIM (https://www.omim.org ).

### Bioinformatics Analyses

A dataset containing the common targets, scilicet NSCLC, or lung cancer relative targets that based on the object miRNA was obtained after comparison. Interaction network was constructed and analyzed by Cytoscape 3.8, and common targets were obtained by Venny 2.1.0. The STRING database was used to establish the protein–protein interaction (PPI) network with *Homo sapiens* as the target organism. To investigate the interactions of target genes and to obtain the parameters of PPI network, the results of STRING database were exported and then modified by Cytoscape (version 3.8) and calculated by NetworkAnalyzer.

To investigate the core mechanism of the pathway and action and biological pathways associated with the common targets, Kyoto Encyclopedia of Genes and Genomes (KEGG) pathway and Gene ontology enrichment analyses were conducted to access the critical information. DAVID Bioinformatics Resources 6.8 (https://david.ncifcrf.gov/) was employed to process the KEGG pathway and GO enrichment analysis of common targets. The acquired terms matched the condition of *p*<0.05. Results visualization were implemented by using R software.

### Statistical Analysis

Plots were produced using GraphPad Prism Version 5.00 (GraphPad Software, San Diego, CA, USA), and data were analyzed by SPSS 15.0 (SPSS, Inc., Chicago, IL, USA). ANOVA was used to analyze the differences between groups, and *t*-test was adopted for two or more independent samples. All reported difference with *p*-values <0.05 were statistically significant.

## Results

### Characterization of Purified Exosomes

**Figure 1:**
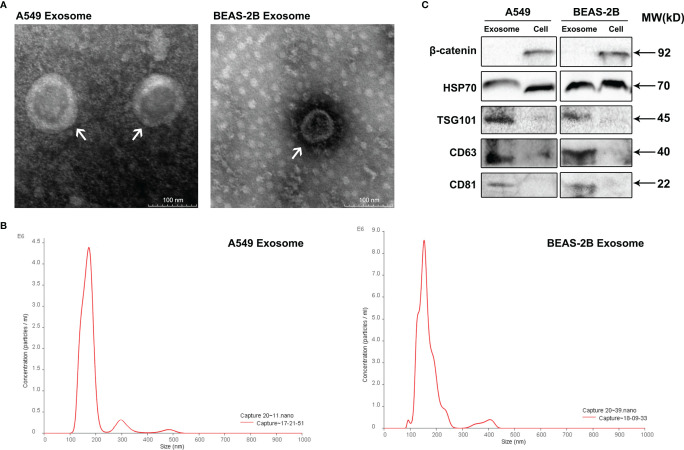
Identification of isolated exosomes from A549 cells and BEAS-2B cells**. (A)** Transmission electron microscopy shows that the isolated exosomes from A549 cells and BEAS-2B cells are vesicle-like; the presented scale is equal to 100 nm. **(B)** The isolated exosomes were measured by NanoSight Technology for size distribution. **(C)** Western blot was applied to verify the expression of related proteins β-catenin, HSP70, TSG101, CD63, and CD81.

Exosomes are small extracellular vesicles (EVs) that are naturally decomposed from cells and whose formation is confined by a lipid bilayer and cannot divide and replicate (8). Exosomes were isolated from A549 cells culture supernatants by differential ultracentrifugation, and then, the characteristics of these vesicles were verified through TEM, NTA, and Western blot analysis. TEM observation revealed that the isolated exosomes showed small cup-shaped circular vesicles, remain intact with a diameter range of approximately 100 nm (**Figure 1A**). NTA showed that vesicles were homogeneous in size with a median number of 150 nm (**Figure 1B**). To further confirm the characterization of purified exosomes, Western blotting assays were carried to detect exosomes marker proteins (HSP70, TSG101, CD63, and CD81) in A549 cells, BEAS-2B cells, and their derived exosomes (**Figure 1C**). As expected, our results showed that the protein expression levels of the tetraspanins protein family CD63 and CD81 and the cytosolic protein family TSG101 were more abundant in exosomes than in cell lysis buffer. We also observed that the negative marker protein β-catenin was attenuated in exosomes (**Figure 1C**). These results support the conclusion that the isolated vesicles in A549 cells and BEAS-2B cells were true exosomes and are available in the follow-up experiments.

### Exosomes From A549 Cells Induced BEAS-2B Cells Migration and Invasion

**Figure 2:**
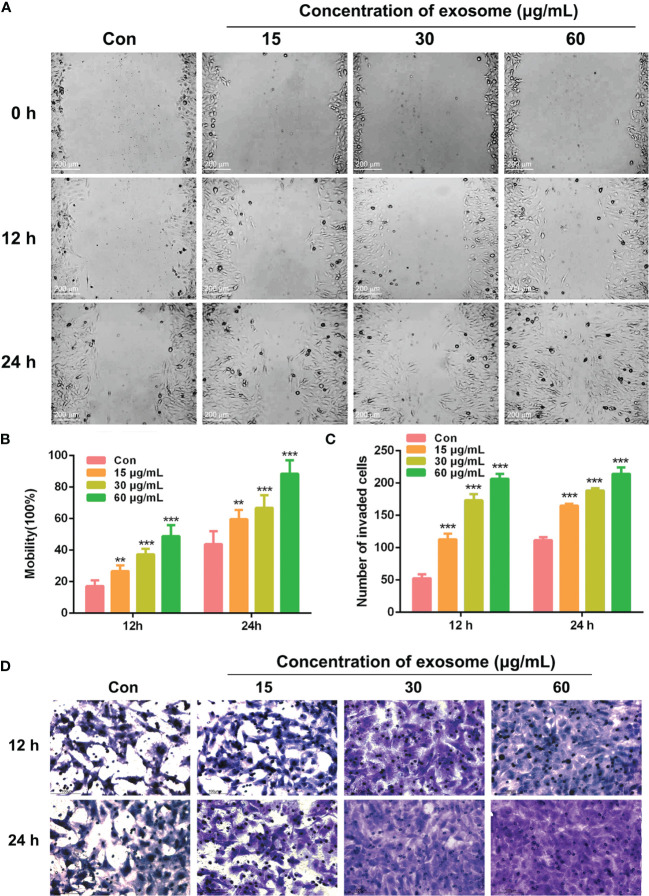
The migration and invasion of BEAS-2B cells after treating with A549-cell-derived exosomes. **(A)** BEAS-2B cell migration induced by different concentrations (0, 15, 30, and 60 μg/ml) of A549-cell-derived exosomes at 0, 12, and 24 h is shown in the wound healing assay. Statistical analysis of scratch distance changes of **(B)** migration and cells number of **(C)** invasion; significant difference between treatment and control groups is indicated as **p < 0.01, ***p < 0.001, and n ≥ 3. **(D)** BEAS-2B cells invasion induced by different concentrations (0, 15, 30, and 60 μg/ml) of A549-cell-derived exosomes at 12 and 24 h is shown in the invasion assay.

Exosomes contain massive bioactive molecules that can be delivered to recipient cells and alter their biological behavior. Several studies have shown that exosomes could induce tumor growth and modify cancer cell microenvironment and then promote the tumor’s multiplication, migration, and invasion (7, 21, 22). The concentration of exosomes was quantified using bicinchoninic acid protein reagent kit. To study the capability of migration and invasion of BEAS-2B cells after treatment with multiple concentrations (0, 15, 30, and 60 μg/ml) of exosomes derived from A549 cells, we executed the wound healing assay and the cell invasion assay. The results indicated that with an increased concentration of A549-cell-derived exosomes, cell migration and invasion (**Figures 2A–D**) of BEAS-2B cells were significantly increased in a time- and dose-dependent manner in wound scrape model and transwell chamber model.

### Exosomes Derived From A549 Cells Modified EMT Marker of BEAS-2B Cells

**Figure 3:**
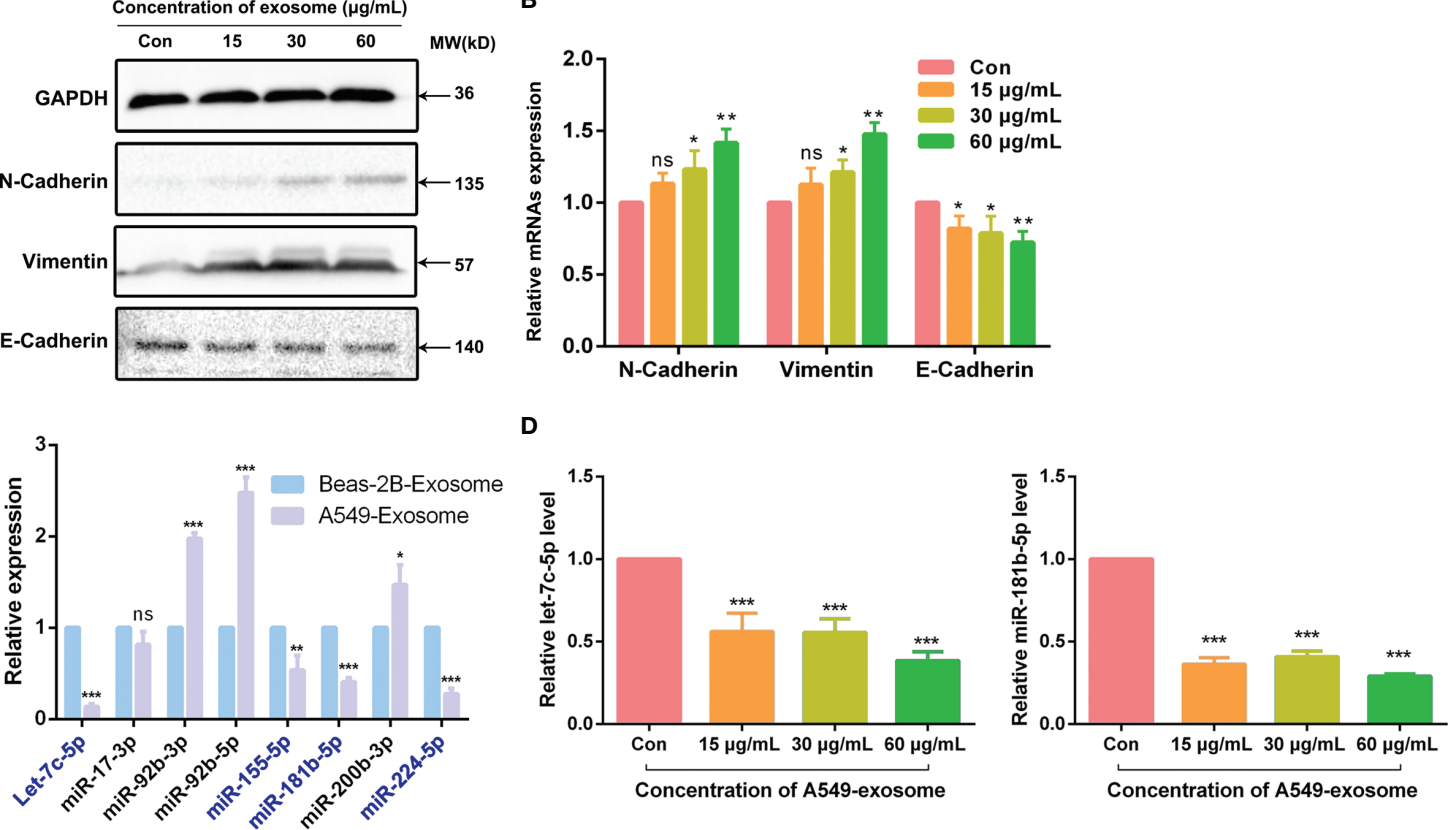
A549-cell-derived exosomes promoted EMT in BEAS-2B cells. **(A)** EMT markers of BEAS-2B cells were detected by Western blot after co-cultured with A549-cell-derived exosomes. **(B)** Relevant mRNAs expression of EMT-specific markers of BEAS-2B cells after co-cultured with A549-cell-derived exosomes were measured by RT-qPCR. **(C)** The expression of let-7c-5p, miR-17-3p, miR-92b-3p, miR-92b-5p, miR-155-5p, miR-181b-5p, miR-200b-3p, and miR-224-5p in exosomes secreted by A549 cells and BEAS-2B cells were compared by RT-qPCR assay. **(D)** The expression levels of let-7c-5p and miR-181b-5p in BEAS-2B cells treated with A549-cell-derived exosomes for 48 h by RT-qPCR. ns represents no significance; significant difference between treatment and control groups is indicated at *p < 0.05, **p < 0.01, ***p < 0.001, and n ≥ 3.

As is known to all, cancer cells could acquire migratory ability through EMT, which is closely related to the initiation of migration and invasion. As exosomes derived from A549 cells could induce migration and invasion in BEAS-2B cells, we also explored whether A549-cell-derived exosomes were involved in the promoting effects of EMT after coculture of the BEAS-2B cells; the changes in EMT biomarkers were tested by Western blotting assay and real-time quantitative PCR (RT-qPCR) assay. As a kind of intercellular adhesion molecules (ICAMs), N-cadherin and E-cadherin are chiefly distributed in mesenchymal and epithelial phenotype, respectively (23, 24). Vimentin is also regarded as a biomarker of EMT and is often overexpressed when cancer cells acquire their mesenchymal phenotype (25). As anticipated, our results showed that the mesenchymal markers N-cadherin and vimentin in BEAS-2B cells were upregulated at both pre- and post-transcriptional levels after treatment with A549-cell-derived exosomes. However, the expression of epithelial marker E-cadherin showed a completely opposite trend (**Figures 3A, B**). All the research shows that exosomes derived from A549 cells modified the expression of EMT makers in BEAS-2B cells.

### Differentially Expressed of miRNAs Between A549 Cells and BEAS-2B-Cell-Derived Exosomes

Total RNAs extracted from A549-cell- and BEAS-2B-cell-derived exosomes were used for miRNA library preparation and sequencing. Library preparation and sequencing were performed at RiboBio (Guangzhou, China) as previously described. The library of miRNA profile was constructed according our group previous work (16). According to the miRNA expression library, we identified that 602 miRNAs were downregulated in exosomes derived from A549 cells compared with BEAS-2B cells, of which 223 cases had statistically differences (*p < 0.05). To verify the miRNA sequencing results, we check out the expression of a number of the identified miRNAs by quantitative real-time PCR assay. The relative expression levels of let-7c-5p, miR-17-3p, miR-92b-3p, miR-92b-5p, miR-155-5p, miR-181b-5p, miR-200b-3p, and miR-224-5p were chosen for further validation (**Figure 3C**). Notably, the expression levels of a total of seven genes were consistent with the sequencing results (**Table 2**). Furthermore, let-7c-5p showed notable fold change of 7.2 between exosomes obtained from A549 cells and BEAS-2B cells.

**Table 2:** Differential expression of miRNAs between A549 and BEAS-2B cells exosomes.

Systematic name of miRNAs	expression level of A compared with B	log2 (A/B)	*p*-value
hsa-let-7c-5p	Down	−1.9865	<0.001
hsa-miR-17-3p	Up	2.6744	<0.05
hsa-miR-92b-3p	Up	1.5005	<0.001
hsa-miR-92b-5p	Up	2.6914	<0.001
hsa-miR-155-5p	Down	−3.5498	<0.001
hsa-miR-181b-5p	Down	−1.1938	<0.001
hsa-miR-200b-3p	Up	2.7494	<0.001
hsa-miR-224-5p	Down	−1.2614	<0.001

### Exosomal miRNA Expression Differ in NSCLC Patients’ Sera

**Figure 4:**
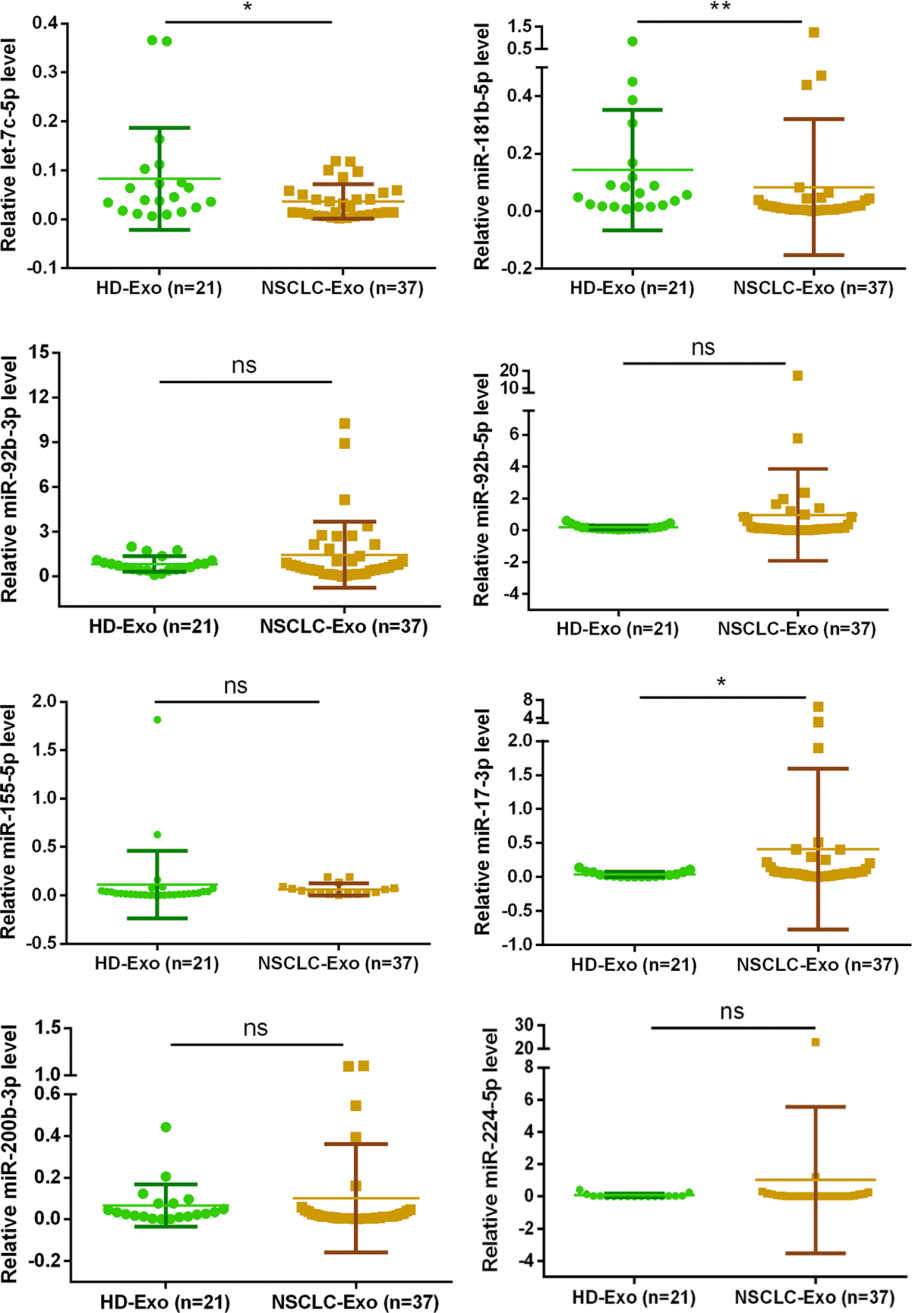
The exosomal miRNA expression in 21 normal persons and 37 NSCLC patients. RT-qPCR was utilized to compare the exosomal miRNA expression level of let-7c-5p, miR-17-3p, miR-92b-3p, miR-92b-5p, miR-155-5p, miR-181b-5p, miR-200b-3p, and miR-224-5p from the serum of healthy controls (n = 21) and NSCLC patients (n = 37). Non-parametric test was adopted to analyze the differences between groups; ns, no significance; *p < 0.05, **p < 0.01, and n ≥ 3.

All patients provided written informed consent before they were included in the research, and this research conformed to the ethical guidelines and was approved by the hospital’s Ethics Committee. The samples were accumulated from 21 healthy persons and 37 NSCLC patients in Second People’s Hospital of DaTong. The expression levels of exosomal miRNA miR-17-3p in the serum of NSCLC patients was notably higher than that of healthy volunteers, while the expression levels of let-7c-5p and miR-181b-5p were opposite (**Figure 4**). Combining these results of clinical samples with that of *in vitro*, the expression levels of let-7c-5p and miR-181b-5p displayed a potential association with the NSCLC, and they had been chosen for further study.

### A549-Cell-Derived Exosomes Downregulate miRNA Expression in BEAS-2B Cells

Since let-7c-5p and miR-181b-5p were decreased in A549-cell-derived exosomes, we were curious about whether A549-cell-derived exosomes could lessen those two miRNAs expression in BEAS-2B cells. To observe the activity of A549-cell-derived exosomes, BEAS-2B cells were coincubated with the exosomes in doses of 0, 15, 30, and 60 μg/ml for 48 h, and then, RT-qPCR assay was performed to monitor the expression level of let-7c-5p and miR-181b-5p. The results showed that the expression of let-7c-5p and miR-181b-5p were significantly decreased in post-treated BEAS-2B cells, and the effect was decrease in a dose-dependent manner (**Figure 3D**).

### Combined Effect of Let-7c-5p and miR-181b-5p on Migration and Invasion of BEAS-2B Cells

**Figure 5:**
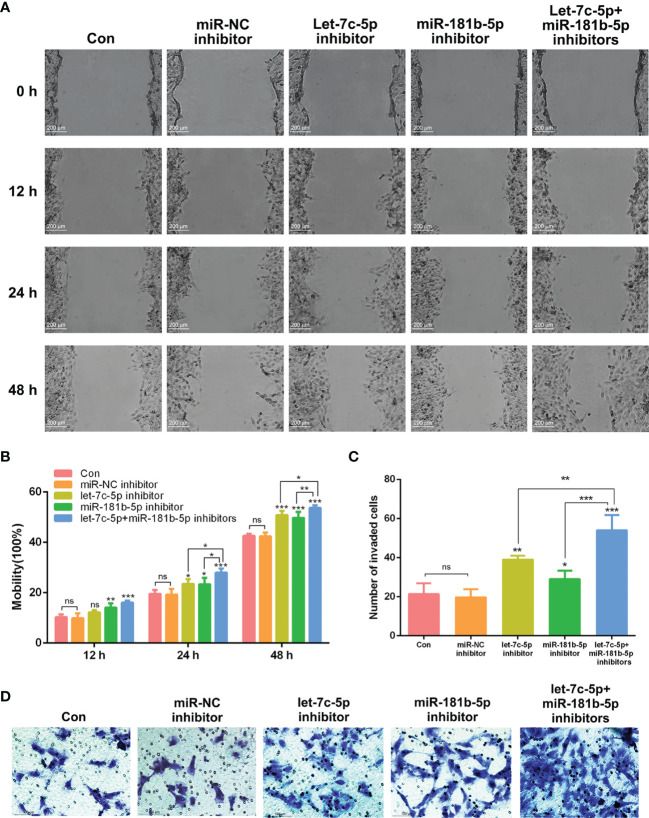
let-7c-5p and miR-181b-5p inhibitors induced the migration and invasion of BEAS-2B cells. **(A)** Wound healing assay was used to identify the migration of BEAS-2B cells that were influenced by let-7c-5p and miR-181b-5p inhibitors for 0, 12, 24, and 48 h. Statistical analysis of **(B)** mobility and **(C)** invasion ratio of BEAS-2B cells treated with let-7c-5p and miR-181b-5p inhibitors. ns represents no significance; *p < 0.05, **p < 0.01, and ***p < 0.001, and n ≥ 3. **(D)** Transwell assay was used to identify the invasion of BEAS-2B cells that were influenced by let-7c-5p and miR-181b-5p inhibitors.

According to the published studies, let-7c-5p and miR-181b-5p were shown to be closely associated with the invasion and migration of cancer (26–28). To verify whether lack of let-7c-5p and miR-181b-5p was critical for promoting the migration and invasion of BEAS-2B cells, miRNA inhibitors were employed to lessen the expression of let-7c-5p and miR-181b-5p in BEAS-2B cells. Migration of BEAS-2B cells showed a slight improvement after transfecting by let-7c-5p and miR-181b-5p inhibitors, respectively, and such improvement was enhanced by the combination of two inhibitors (**Figures 5A, B**). Invasive capacity of BEAS-2B cells was evaluated by transwell experiment, and it turned out that BEAS-2B cells were more invasive with reduction in both let-7c-5p and miR-181b-5p (**Figures 5C, D**).

### Overexpression of let-7c-5p and miR-181b-5p Attenuated the Effect of A549-Cell-Derived Exosomes on BEAS-2B Cells

**Figure 6:**
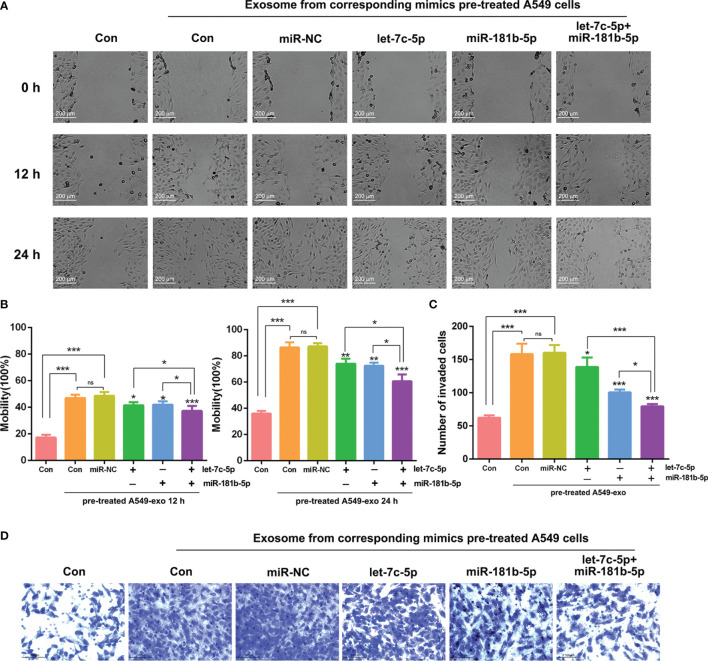
Impact of exosomes from different treatments of A549 cells on migration and invasion of BEAS-2B cells. **(A)** Wound healing assay was used to identify the migration of BEAS-2B cells that were influenced by exosomes derived from let-7c-5p/miR-181b-5p mimics pre-treated A549 cells for 0, 12, and 24 h. Statistical analysis of **(B)** mobility and **(C)** invasion ratio of BEAS-2B cells treated with let-7c-5p or miR-181b-5p high-expression exosomes, ns, no significance, *p < 0.05, **p < 0.01, and ***p < 0.001, and n ≥ 3. **(D)** Transwell assay was used to identify the invasion of BEAS-2B cells that were influenced by exosomes from let-7c-5p or miR-181b-5p mimics-pretreated A549 cells. ns, no significance.

The results above revealed that the attenuation of let-7c-5p and miR-181b-5p had a critical effect in cell migration and invasion. The exosomes derived from A549 cells could induce BEAS-2B cell migration, invasion, and EMT, and they also possessed poor expression of miRNA let-7c-5p and miR-181b-5p. To gain more evidence that let-7c-5p and miR-181b-5p are the key factors that induce the invasion of BEAS-2B cells, rather than other pathways, we built a let-7c-5p and miR-181b-5p overexpression model in cells to monitor its migration and invasion activities. Exosomes were extracted after let-7c-5p and miR-181b-5p mimics were transfected into A549 cells, respectively, or in combination, and then, those exosomes were added into BEAS-2B cells and co-incubated for 12 or 24 h. Mobility of BEAS-2B cells was weakened after incubation with let-7c-5p or miR-181b-5p overexpression exosomes compared to that with regular A549-cell-derived exosomes in the wound healing assay. Overexpressed let-7c-5p and miR-181b-5p both exhibited stronger attenuation of BEAS-2B cells mobility in a time-dependent manner (**Figures 6A, B**). As shown in transwell assay, the invasion of BEAS-2B cells was assessed, and the results were consistent with wound healing assay (**Figures 6C, D**). Hence, the restoration effect of let-7c-5p and miR-181b-5p overexpression in combination surpassed that when they function separately in A549 cells.

### Target mRNA Prediction of let-7c-5p and miR-181b-5p

**Figure 7:**
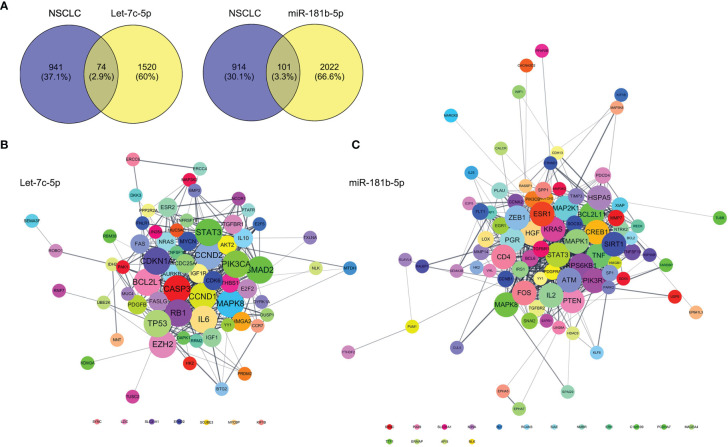
Prediction of let-7c-5p and miR-181b-5p target gene. **(A)** The common targets of retrieval NSCLC relative targets compared with the predicted miRNA targets of let-7c-5p and miR-181b-5p, respectively. The PPI network with the common targets of **(B)** let-7c-5p and **(C)** miR-181b-5p with NSCLC.

Targets genes of let-7c-5p and miR-181b-5p were predicted by TargetsScan, PicTar, and miRDB databases, respectively. The total number of target genes of let-7c-5p was 990 from miRDB, 602 from PicTar, and 1,191 from TargetsScan; after removing the duplicate values, a total of 1,594 predicted target genes were recruited. The total number of 2,123 miR-181b-5p target genes was obtained from the identical databases after removing the duplicate values; the specific predicted target numbers were 1,408, 428, and 1,367 for miRDB, PicTar, and TargetsScan. To explore the relationship of target gene between object miRNA and NSCLC, the OMIM, DisGeNet, TTD, and GeneCards database were employed to retrieve NSCLC or lung cancer-related targets. A total of 190, 31, 158, and 941 targets were collected from the above databases, respectively. Then, the results were integrated, after which the reduplicative data were removed. The retrieved NSCLC relative targets with a total of 1,016 were compared with the predicted miRNA targets of let-7c-5p and miR-181b-5p separately; then, 74 and 101 common targets, respectively, were collected (**Figure 7A**). These common targets became the essential study objects that represent the potential significance of the regulation mechanism of miRNA in NSCLC.

### Investigation of the Regulation Mechanism of let-7c-5p and miR-181b-5p in NSCLC by Bioinformatics Analyses

**Figure 8:**
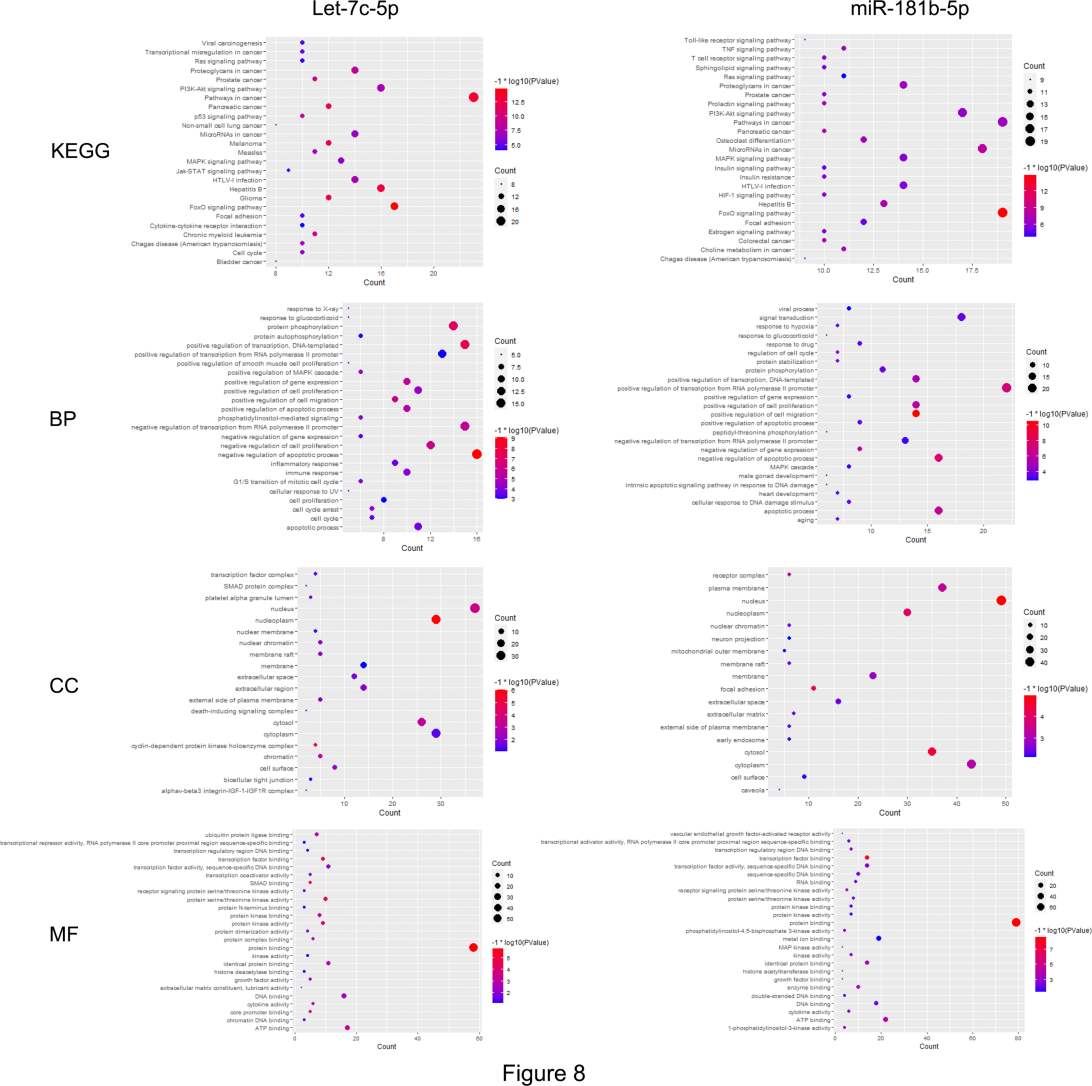
The common targets analyzed by KEGG and GO enrichment. Results of KEGG and GO (including BP, CC, MF) enrichment analysis for the targets gene of let-7c-5p and miR-181b-5p, respectively (*p* ≤ 0.05).

First, the STRING database was used to create a network consisting of common targets. To understand how the multiple targets function in NSCLC, the PPI networks become an indispensable research method. The PPI network that was construed with the common targets of let-7c-5p (miR-181b-5p) and NSCLC is shown in **Figures 7B, C**. The target genes with a higher node degree than 20 were regarded as the critical targets influenced by miRNA. In other to find out the regulation mechanism of let-7c-5p and miR-181b-5p in NSCLC, the common targets were analysis by KEGG and GO enrichment using DAVID. Results of the enrichment were visualized by R software by way of a bubble diagram (**Figure 8**). Eighty-two results were returned by KEGG enrichment for let-7c-5p and 80 for miR-181b-5p. Among the 217 terms from GO analysis of let-7c-5p, 164 terms turned out to be involved in biological processes (BPs), 20 in cellular component (CC), and 33 in molecular functions, while among the 300 terms for miR-181b-5p, the number of terms were 221, 31, and 48, respectively. Results of KEGG pathway analyses indicated that let-7c-5p and miR-181b-5p possessed the coefficient in MAPK signaling pathway, RAS signaling pathway, FoxO signaling pathway, PI3K-Akt signaling pathway, and Focal adhesion. Critical nodes of let-7c-5p were mainly implicated in MAPK signaling pathway, p53 signaling pathway, transcriptional misregulation in cancer, and focal adhesion. In addition, MAPK signaling pathway, focal adhesion, and Ras signaling pathway were associated with the critical node of miR-181b-5p. As the results showed, focal adhesion and MAPK signaling pathway were essential for the synergy of let-7c-5p and miR-181b-5p in restriction of the EMT. Taking the GO enrichment results together, the number of genes and *p-*value were critical parameters to filter the term of BPs, CC, and molecular function (MF). A larger number of critical genes were utilized as the filter conditions to screen the common BP, CC, and MF of let-7c-5p and miR-181b-5p involved in NSCLC. The synergistic BP function was the response to glucocorticoid; the synergistic CC function included cell surface, the outside of the plasma membrane, extracellular space, membrane, and membrane raft; and the synergistic MF function included cytokine activity, receptor signaling protein serine/threonine kinase activity, and protein kinase activity.

## Discussion

It is widely known that exosomes have a pivotal effect on intercellular communication, especially of tumor progress, including tumorigenesis, angiogenesis, tumor metastasis, invasion, and drug resistance (29, 30). Enriched in tumor microenvironment or circulation, tumor-cell-derived exosomes are easily internalized by neighboring cells or distal cells and then function as a promoter to trigger tumor initiation and metastasis (31, 32). Thus, the influence of A549-cancer-cell-derived exosomes on BEAS-2B normal cells was investigated in our study. After being identified by a serious assay, exosomes that were derived from A549 cells were added to BEAS-2B cells to evaluate the migration and invasion capacity. Results had shown that A549-cell-derived exosomes could facilitate BEAS-2B cell migration and invasion, even EMT (**Figures 2**, **3**). Next, we had a rigorous study on the key bioactive molecules in exosomes.

Exosomes belong to biological vesicles that contain various bioactive molecules, such as proteins, nucleic acid, and lipid, which can be delivered into the recipient cells to endow the specificity (33, 34). The characteristics of the vesicles were verified through TEM, NTA, and Western blot analysis. All EVs bear proteins associated with the biological membrane. To demonstrate the presence of a lipid bilayer in the material analyzed, at least one transmembrane or GPI-anchored extracellular protein must be detected (35). A total of three positive protein markers (including at least one-transmembrane/lipid-bound protein–cytosolic protein) and one negative protein marker of EVs were detected during our identification (**Figure 1C**). Characterization of single vesicles was conducted by two different but complementary techniques: electron microscopy and single particle analyzers (**Figures 1A, B**).

miRNAs play a crucial role in various of biological processes, which include the regulation of cell development, differentiation, proliferation, and apoptosis. Functional studies have confirmed that multifarious miRNAs molecules are in contact with tumor genesis and development, and miRNAs have functions similar to oncogenes or tumor suppressor genes (36, 37). According to our previous work (16), miRNA profile library of exosomes derived from A549 cells and BEAS-2B cells were constructed. Eight exosomal miRNAs were chosen from the differential expression miRNA for further verification, two of which (let-7c-5p, miR-181b-5p) showed significant difference not only between cancer cells and normal cells but also between NSCLC patients and healthy person.

let-7 is one of the first miRNAs discovered in *Caenorhabditis elegans*, and its biological functions are highly conserved from *C. elegans* to humans (38). As a major regulator of differentiation, pluripotency, and apoptosis, let-7 family possesses a variety of physiological functions, including growth, development, and cell adhesion, and especially serve as a tumor suppressor (39). During the tumorigenesis and the tumor progress, let-7 possesses unlimited potentialities as a bioactive molecule for diagnosis, therapy, and prognosis (40). let‐7c, miR‐145, and miR‐221 showed decreased expression in patients with low‐risk prostate cancer, and therefore, those miRNAs have the potential as diagnosis biomarkers (41). Jilek et al. illuminated that liposomal-polyplex (LPP)-formulated biological let-7c may serve as a safe and powerful therapeutic strategy for hepatocellular carcinoma (42). The let-7c miRNA cluster expression could be detected by RT-qPCR, as a responsible non-invasive clinical instrument to improve prognosis assessment of high-grade non-muscle-invasive bladder cancer, in the urinary tract (43). In addition, let-7c-5p is still highly relevant to the tumorigenesis and migration of malignant tumor (44, 45).

As members of the highly conserved miRNA family in different species, miR-181b plays prominent roles in various biological process, even different diseases. Particularly, it is well known as a cancer suppressor that dysregulates numerous cancers (46). MiR-181b becomes a potential candidate for diagnosis and treatment on account of dysregulation in different kinds of cancer, including lung cancer, gliomas, gastric cancer, and colorectal cancer (47–50). Apart from having attenuation effect in lung cancer, miR-181b was further identified to have affinity to chemoresistance (51, 52). Recent research has reported that tumor-cell-derived exosomal miR-181b-5p improves tumorigenesis and metastasis of esophageal squamous cell carcinoma by inducing angiogenesis, showing that the exosomal miRNA floating in a tumor microenvironment serves as a promoter of tumor progression (53).

Numerous current research have proved that exosomes secreted by donor cells can transmit bioactive substance to neighboring cells to influence their characteristic and reprogram the phenotypes of recipient cells, with the potential to modulate carcinogenic consequences (54, 55). Exosomes derived from HQ-transformed malignant cells were proven to be able to transmit miR-221 to normal recipient cells to promote cell viability (56). Exosomes isolated from patients with sepsis lung injury were proven to cause lung bronchial epithelial cell injury through miR-1298-5p and its target SOCS6 *via* regulating STAT3 signaling pathway (57).

To further investigate whether aberrantly expressed miRNA could be delivered from donor cells to recipient cells, attenuated expression of let-7c-5p and miR-181b-5p was detected by RT-qPCR after co-incubation with A549-cell-derived exosomes for 12 h. In addition, the combination of let-7c-5p and miR-181b-5p inhibitors significantly induced the migration or invasion of BEAS-2B cells more than either alone, which indicated that let-7c-5p and miR-181b-5p may have the common target mRNA that is related to mobility and invasiveness (**Figures 5**, **6**). Recent studies had illuminated that let-7c acts as a tumor suppressor that inhibits the tumor migration and invasion in NSCLC, hepatocellular carcinoma, and glioma (58–60). Liu et al. reported that increased expression of miR-181a/b induces the proliferation activities, tumor invasion, and metastasis of neuroblastoma cells by targeting ABI1 (46). To verify the vital role of exosomal let-7c-5p and miR-181b-5p, the exosomes from A549 cells pre-treated with let-7c-5p and miR-181b-5p mimics were added to BEAS-2B cells, respectively or in combination; consequently, the influence of cell migration and invasion showed a significant reduction.

Bioinformatics analyses were adapted to explore in-depth the synergistic effect of let-7c-5p and miR-181b-5p. The critical node with degrees larger than 20 was calculated and obtained after common targets were constructed and analyzed by STRING and Cytoscape. Results of KEGG pathway analyses revealed that focal adhesion and MAPK signaling pathway were essential for the synergy of let-7c-5p and miR-181b-5p in the restriction of the EMT (**Figures 7**, **8**). Cell adhesion to ECM depends on integrin-mediated linkage to extracellular ECM molecules and intracellular cytoskeleton, and the intracellular domain is linked to the cytoskeleton through intracellular focal adhesions as demonstrated (61). Focal adhesions not only offer physical attachment of cells to the ECM through the integrin receptor but also initiate signaling cascades that regulate cell proliferation, migration, and survival (62). As a key signal pathway of cell adhesion and migration, focal adhesion was highly relevant to the invasion and migration of tumor (63–65). Numerous studies had disclosed the relationship between MAPK signal pathway and EMT, such as Musashi2 promotes EMT in pancreatic cancer through ZEB1-ERK/MAPK, Integrin/EGFR-ERK/MAPK, and ERK/MAPK signaling pathway (66–68). MAPK signal pathway was also associated with tumor proliferation and metastasis in cervical cancer (69). The enrichment indicated that MAPK8 was targeted by let-7c-5p and miR-181b-5p simultaneously and also took part in both focal adhesion and MAPK signaling pathway, which means that MAPK8 might be the essential target gene of the synergistic effect in the EMT inhibition (**Figures 7**, **8**).

## Conclusion

In summary, we illustrated that the BEAS-2B cells acquire malignancy characteristic by endocytosing the A549-cell-derived exosomes, including migration, invasion, and EMT. The pivotal biomolecules of this progress showed to be exosomal let-7c-5p and miR-181b-5p. The attenuation of let-7c-5p and miR-181b-5p facilitated the mobility of BEAS-2B cells and promoted the cells developing into malignant tumors. Bioinformatics analyses had revealed that focal adhesion and MAPK signaling pathway are essential for the inhibition of EMT by let-7c-5p and miR-181b-5p. Thus, let-7c-5p and miR-181b-5p possess the mass potential for the early diagnosis of lung cancer.

## Data Availability Statement

The original contributions presented in the study are included in the article/supplementary material. Further inquiries can be directed to the corresponding authors.

## Ethics Statement

The studies involving human participants were reviewed and approved by the Second People’s Hospital of Datong. The patients/participants provided their written informed consent to participate in this study. Written informed consent was obtained from the individual(s) for the publication of any potentially identifiable images or data included in this article.

## Author Contributions

J-YZ, Y-QW, and J-JF conceived the study and revised this manuscript. YL, C-YS, and Y-YY performed the experiments and wrote the manuscript. JW and J-JL analyzed the data. All authors approved the manuscript.

## Funding

This work was supported by the National Key R&D Program of China (2021YFE0202000), the National Natural Science Foundation of China (81903467 and 81902152), Fund of Guangdong Education Department (2021ZDZX2006 and 2020KTSCX102), and Scientific and Technological Innovation Programs of Higher Education Institutions in Shanxi (2020L0483).

## Conflict of Interest

The authors declare that the research was conducted in the absence of any commercial or financial relationships that could be construed as a potential conflict of interest.

## Publisher’s Note

All claims expressed in this article are solely those of the authors and do not necessarily represent those of their affiliated organizations, or those of the publisher, the editors and the reviewers. Any product that may be evaluated in this article, or claim that may be made by its manufacturer, is not guaranteed or endorsed by the publisher.
